# An Empirical Evaluation of the Utility of Convex Hull and Standard Ellipse Areas for Assessing Population Niche Widths from Stable Isotope Data

**DOI:** 10.1371/journal.pone.0056094

**Published:** 2013-02-06

**Authors:** Jari Syväranta, Anssi Lensu, Timo J. Marjomäki, Sari Oksanen, Roger I. Jones

**Affiliations:** Department of Biological and Environmental Science, University of Jyväskylä, Jyväskylä, Finland; University of California Berkeley, United States of America

## Abstract

Stable isotope analyses are increasingly employed to characterise population niche widths. The convex hull area (TA) in a δ^13^C–δ^15^N biplot has been used as a measure of isotopic niche width, but concerns exist over its dependence on sample size and associated difficulties in among-population comparisons. Recently a more robust method was proposed for estimating and comparing isotopic niche widths using standard ellipse areas (SEA), but this approach has yet to be tested with empirical stable isotope data. The two methods measure different kind of isotopic niche areas, but both are now widely used to characterise isotopic niche widths of populations. We used simulated data and an extensive empirical dataset from two fish populations to test the influence of sample size on the observed isotopic niche widths (TA and SEA). We resampled the original datasets to generate 5000 new samples for different numbers of observations from 5 to 80 to examine the statistical distributions of niche area estimates for increasing sample size. Our results illustrate how increasing sample size increased the observed TA; even sample sizes much higher than *n* = 30 did not improve the precision for the TA method. SEA was less sensitive to sample size, but the natural variation in our empirical fish δ^13^C and δ^15^N data still resulted in considerable uncertainty around the mean estimates of niche width, reducing the precision particularly with sample sizes *n*<30. These results confirm that the TA method is less appropriate for estimating population isotopic niche areas using small samples, especially when considerable population level isotope variation is expected. The results also indicate a need for caution when using SEA as a measure of trophic niche widths for consumers, particularly with low sample sizes and when the distribution and range for population isotope values are not known.

## Introduction

Population niche is an important concept in ecology for understanding species interactions and the structuring of communities. Hutchinson [1] considered niche an ‘*n*-dimensional hypervolume’ defined by all the resources exploited by a population. In practise such a volume is impossible to quantify; potentially more tractable is the feeding niche (or trophic niche), which refers to the dietary diversity of an animal [2]. Traditional measures of trophic niche width from gut contents analysis have required laborious examinations of the diets of many individuals in a population, and preferably over an extended time period to take account of temporal fluctuations in diet.

More economical and integrative measures of animal diets can potentially be obtained by stable isotope analysis (SIA). The ratios of stable isotopes of carbon and nitrogen (^13^C/^12^C and ^15^N/^14^N, expressed relative to a standard as δ^13^C ‰ or δ^15^N ‰) provide time-integrated information of assimilated diet and, due to various technical developments over recent decades, these analyses are now routinely available to ecologists. SIA can be used to assess both diet sources, since δ^13^C values of consumers closely match those in their diet [3],[4], and trophic position in the food chain due to consistent increase in δ^15^N values (typically 3–4‰ per trophic level) of consumers higher in the food chain [5],[6]. In addition, the δ^13^C and δ^15^N values can often integrate information on habitat utilisation, since their values can vary significantly depending on the habitat [7]. Hence SIA offers an appealing method to characterise niche widths of animals.

Bearhop *et al.* [8] proposed a practical approach to assess feeding niche width using variances associated with population mean δ^13^C and δ^15^N values. More recently, Layman *et al.* [9] proposed the use of community-wide metrics, such as convex hull areas, to analyse food web structure from SIA data, while Schmidt *et al.* [10] introduced circular statistics for analysing stable isotope food web data. Although these methods have generated criticism [11],[12], several authors have nevertheless later applied community-wide metrics (*sensu* [9]) to assess population niche width (or “isotope niche width”) from individual δ^13^C and δ^15^N data (e.g. [13]–[16], Appendix S1). When stable isotope data from many individuals within a population are presented as a δ^13^C–δ^15^N biplot, this is essentially a two-dimensional representation of the feeding niche of the species, in which data points correspond to individual diets expressed as paired isotopic coordinates. The size of the isotope niche can then be calculated as the minimum convex polygon (or convex hull), i.e. the smallest area encompassing all the observations.

**Figure 1:**
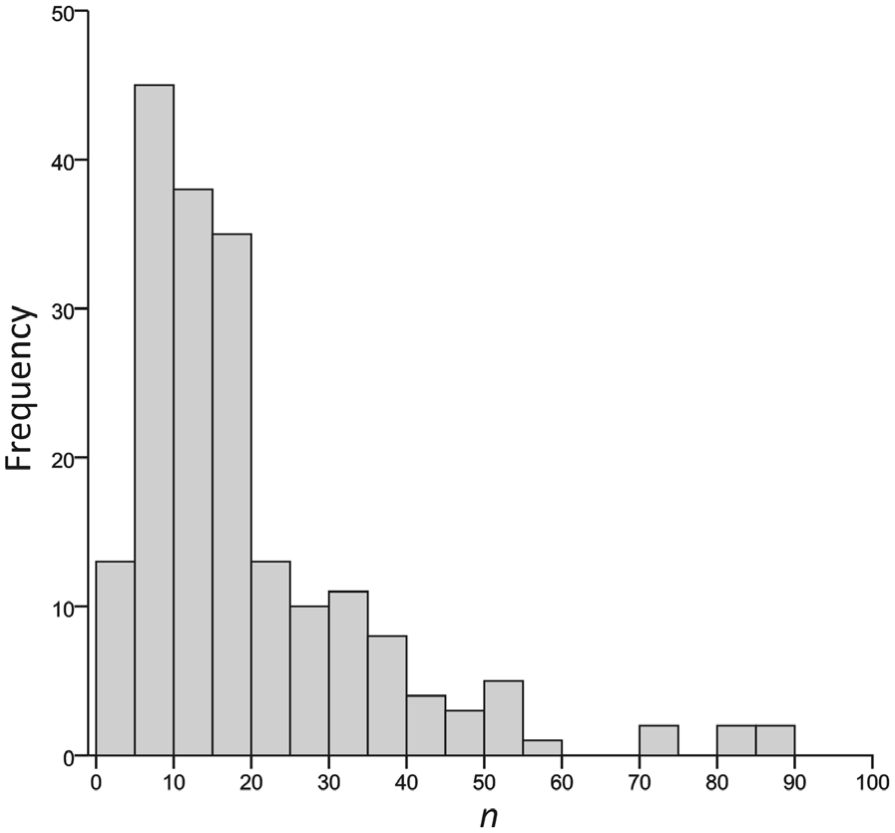
Frequency distribution of sample sizes for estimating population niche widths in published studies using stable isotope methods (TA or SEA). The data were sourced through literature search in ISI Web of Knowledge and Scopus and publications are listed in Appendix S1.

However, the convex hull area is likely to be highly sensitive to the number of observations [17],[18] and only rarely have stable isotope studies analysed more than 15–20 individuals from a given population for δ^13^C and δ^15^N. Yet studies have shown that within-population variation in δ^13^C and δ^15^N values can be substantial [19]–[22], often with skewed and peaked frequency distributions. Therefore isotopic niche widths estimated from small sample sizes may result in significant underestimations of the true population niche width. Moreover, comparing estimates of niche widths from populations with unequal sample sizes will easily result in flawed conclusions, if the number of analysed individuals dictates the observed niche width. Recently, Jackson *et al.* [18] proposed a promising new method to estimate isotopic niche widths using standard ellipse areas which may not be so sensitive towards sample size. However, they only tested the method using a simulated dataset with an assumed multivariate normal distribution. While some natural communities may indeed exhibit normal distributions in their δ^13^C and δ^15^N values, the literature shows that this is certainly not always true and many aquatic populations in particular seem prone to non-normality in their stable isotope values [20]–[22]. Despite analytical costs having considerably decreased over the last decade, many stable isotope studies still suffer from sample sizes which are too small to allow robust testing for normality; most published studies involve sample sizes <20 (Fig. 1).

Here we test the influence of increasing sample size on the estimate of isotopic niche width, measured as the convex hull total area (TA), standard ellipse area (SEA) and standard ellipse area corrected for small sample sizes (SEA_c_), using an unusually large empirical data set of individuals from two fish populations, perch (*Perca fluviatilis* L.) and roach (*Rutilus rutilus* L.) from a single lake. We complement these analyses with a simulated data set that meets the assumptions on multivariate normality but otherwise replicates the range, variation and covariance observed in the empirical data. We adapted a rarefaction method with bootstrapping and randomly sampled an increasing number of individuals from empirical and simulated fish populations for calculating the niche area with each metric. This method allowed us to efficiently model the likelihood of obtaining a certain niche area with a given sample size. Our aims were to test i) how sensitive the metrics (TA, SEA, SEA_c_) are towards sample size using both empirical and simulated data and ii) how the variation in δ^13^C and δ^15^N values of natural populations and potential violations of assumptions on data normality translate into variation and uncertainty in the niche width estimates. In this way we hope to provoke further discussion about the reliability, benefits and overall utility of these newly popular isotope metrics.

## Materials and Methods

Lake Jyväsjärvi (62° 14′ N, 25° 46′ E) is a moderately eutrophic (total phosphorus concentration around 35–40 µg L^−1^) urban lake in central Finland with an area of 3.4 km^2^. Jyväsjärvi is recovering from earlier severe pollution by municipal and industrial waste waters and recent restoration methods have included mass removals of small fish. Perch and roach analysed for this study were collected from these fish removal catches in 2005 and 2006 allowing a random sampling of 202 perch (mean ± SD total length 124±32 mm, range 57–212 mm) and 173 roach (171±73 mm, 57–275 mm) individuals for SIA. A small muscle sample was dissected from each fish, dried in an oven at 60 °C and ground into homogenous powder using a mortar and pestle. A small subsample (0.6 mg) was then accurately weighed into a tin cup for the analysis of δ^13^C and δ^15^N in a FlashEA 1112 elemental analyzer coupled to a Thermo Finnigan DELTA^plus^ Advantage mass spectrometer (Thermo Electron Corporation, Waltham, MA, U.S.A.) at the University of Jyväskylä following standard protocols.

Reliable testing for the influence of increasing sample size on the TA, SEA and SEA_c_ in a δ^13^C–δ^15^N biplot requires extensive data sets, which are not often available in published ecological stable isotope studies. The 202 perch and 173 roach individuals from Jyväsjärvi were assumed to sufficiently reflect the statistical distributions for the δ^13^C and δ^15^N values of these populations and, therefore, could be used for analysing the effect of sample size on TA, SEA and SEA_c_ by bootstrapping (resampling). 5000 random samples of *n* individuals were drawn from the dataset with replacement and all three metrics were calculated for each draw. The minimum *n* was set to 5 and maximum to 80 (<50 % of the number of individuals in the original datasets). The same procedure was repeated for the simulated data sets, which were generated for both perch and roach such that sample sizes matched the true data sets. Their sample means were the same as those obtained from the true datasets and the covariance structures of the generated δ^13^C and δ^15^N values were the same as in the original data, but the observations followed a multivariate normal distribution.

The metric areas were calculated using a recently published Stable Isotope Bayesian Ellipses in R (SIBER) package [18] for R v.2.10.1 [23]. The original script was modified to include bootstrapping of 5000 isotopic niche areas for each sample size *n*, which were then stored and their distributions examined using percentile values. The simulated data sets were generated with Multivariate Normal and t Distributions (mvtnorm) package in R.

## Results

**Figure 2:**
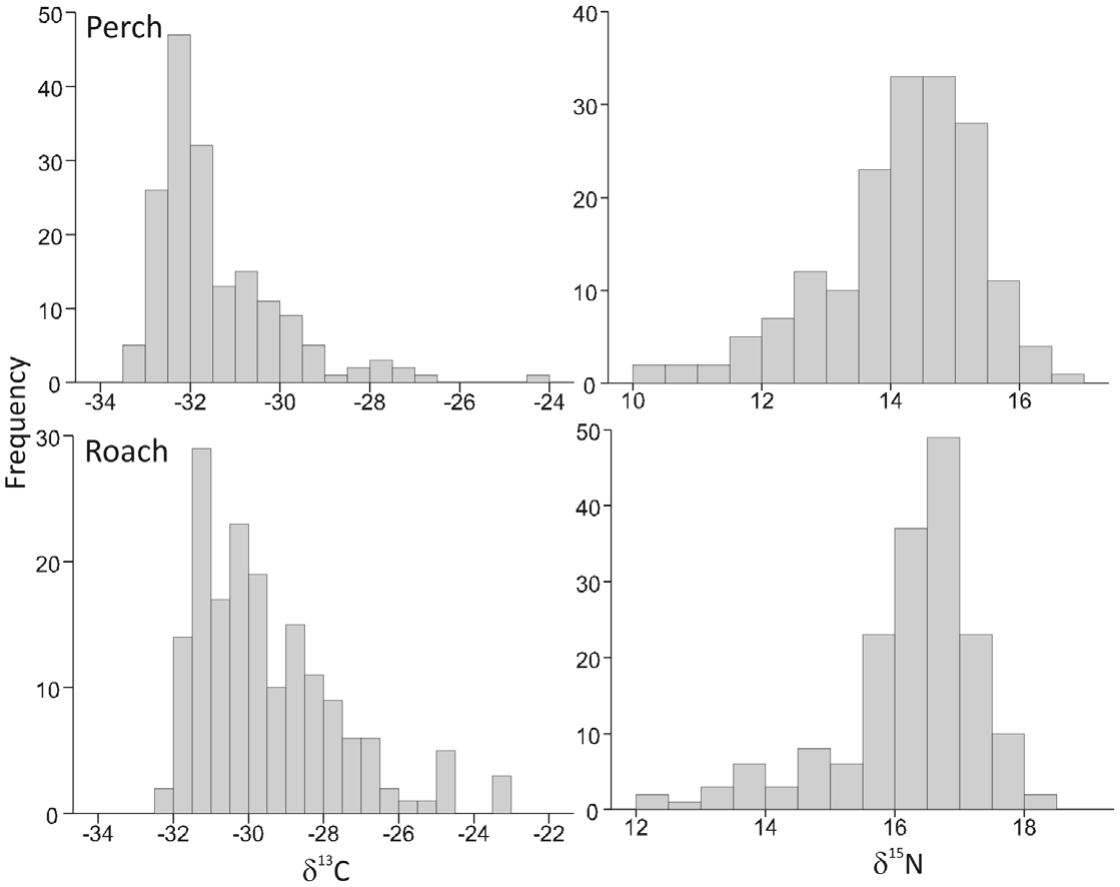
Frequency distributions for δ^13^C and δ^15^N values in empirical data set used in this study. Isotope values of perch (upper panels) and roach (lower panels) populations from Lake Jyväsjärvi showing their peaked and skewed distributions (Skewness: perch δ^13^C = 1.896, δ^15^N = −1.443; roach δ^13^C = 1.162, δ^15^N = −0.883 Kurtosis: perch δ^13^C = 4.816, δ^15^N = 2.501; roach δ^13^C = 1.239, δ^15^N = 0.746).

**Figure 3:**
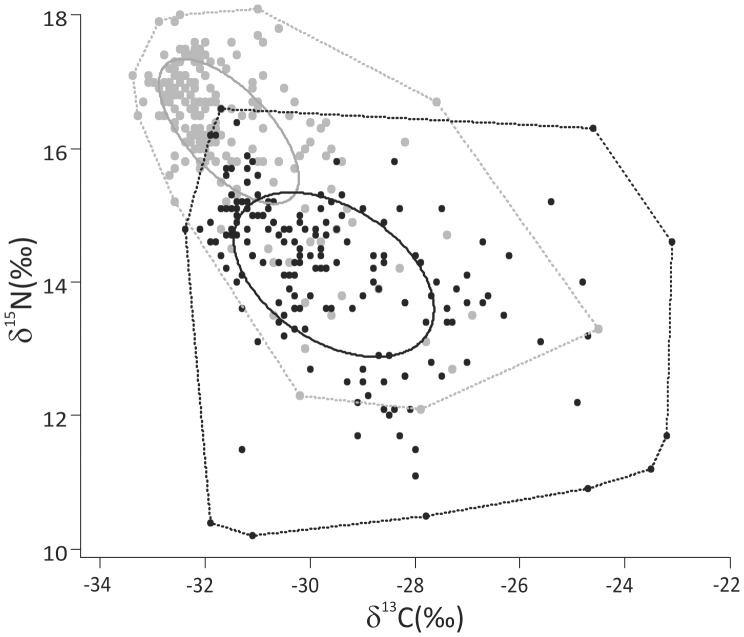
Perch and roach isotope niche width estimates from Lake Jyväsjärvi. Standard ellipse areas (SEA, solid lines) and convex hull TA (dashed lines) are estimated for perch (grey symbols and lines) and roach (black symbols and lines) populations using SIBER [18].

Carbon and nitrogen stable isotope ratios of individual perch and roach exhibited considerable variation, which is not exceptional for natural populations. The frequency distributions of the perch and roach population δ^13^C and δ^15^N values were markedly peaked and skewed (Fig. 2), indicating strong and asymmetric clustering of observations within the δ^13^C–δ^15^N matrix. Moreover, none of the distributions followed a normal distribution, or could be transformed to fit a normal distribution using any common transformation method such as log, arcsine square root or Box-Cox transformations. The TA calculated for the perch (31.7) and in particular the roach (51.1) populations spanned a wide range of both δ^13^C and δ^15^N values (Fig. 3). In contrast, the SEA and SEA_c_ values seemed to be less dependent on the most extreme points and were 3.5 for perch and 6.7 for roach (Fig. 3), overall representing a similar difference between the isotopic niche widths as the TA.

**Figure 4:**
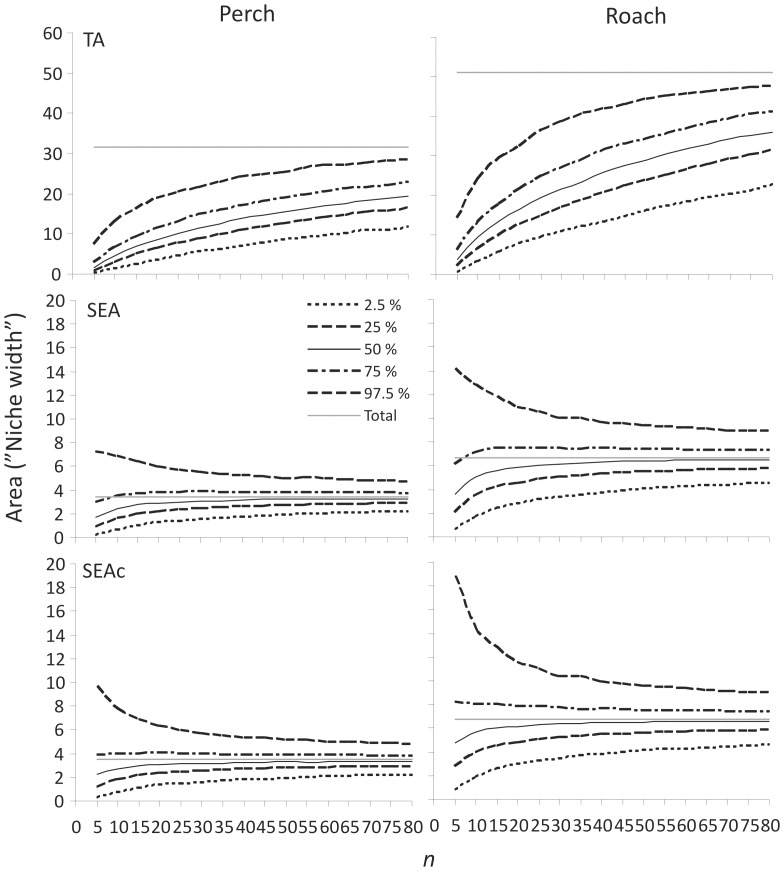
Population isotopic niche width modelling using increasing sample size. Perch (left panel) and roach (right panel) population datasets were used to estimate the convex hull TA, SEA and SEA_c_ calculated for 5000 random selections of individuals with increasing sample size (*n*+5). Lines represent the upper 97.5%, 75%, 50% and lower 25% and 2.5% percentiles for the niche area estimates after each 5000 resamplings with increasing sample size. The solid grey line indicates the observed “true” total niche area for each metric (*n* = 202 for perch and *n* = 173 for roach).

Resampling our datasets using different sample sizes revealed a strong dependence between the sample size and the observed TA (Fig. 4), highlighting the sensitivity of the convex hull TA method towards small and/or unequal sample sizes. Moreover, the natural variation associated with our perch and roach δ^13^C and δ^15^N values resulted in a wide percentile range in the bootstrapped TA niche areas, and consequently the likelihood of obtaining very different inferred niche area estimates with a given sample size was alarmingly high (Fig. 4). Sample sizes of 15–20 individuals reached median (50%) niche areas of 6.8 and 8.6 for perch population when the observed niche area for the whole 202 individuals was as large as 31.7. Moreover, 95% of observations ranged between 2.6–16.7 (*n* = 15) and 3.4–18.8 (*n* = 20) for perch and 5.7–29.6 and 7.8–32.6 for roach, respectively, giving little confidence for the precision of the TA niche area estimates with such sample sizes.

SEA, and in particular SEA_c_, metrics with adequate sample sizes were less biased at estimating the isotopic niche width and were less sensitive to the sample size (Fig. 4). The median of the observations underestimated the niche area with sample sizes 5–10 but approached, and for SEA_c_ matched, the observed total SEA/SEA_c_ metric values with *n*>30. Nevertheless, the resampling method still revealed rather wide percentile ranges also for the SEA and SEA_c_ metrics, although unlike TA the uncertainty decreased quickly with increasing sample size (>30). However, with sample sizes of 15 and 20 individuals, 95% of the SEA values still ranged between 1.0–6.4 and 1.2–6.0 for perch and 2.4–11.8 and 2.8–10.9 for roach, while the observed values for the whole data were 3.5 for perch and 6.7 for roach. Similarly, the SEA_c_ values ranged between 1.0–6.9 and 1.3–6.3, and 2.6–12.8 and 3.0–11.5, respectively (SEA_c_ for the whole data were the same as SEA, i.e. 3.5 and 6.7).

**Figure 5:**
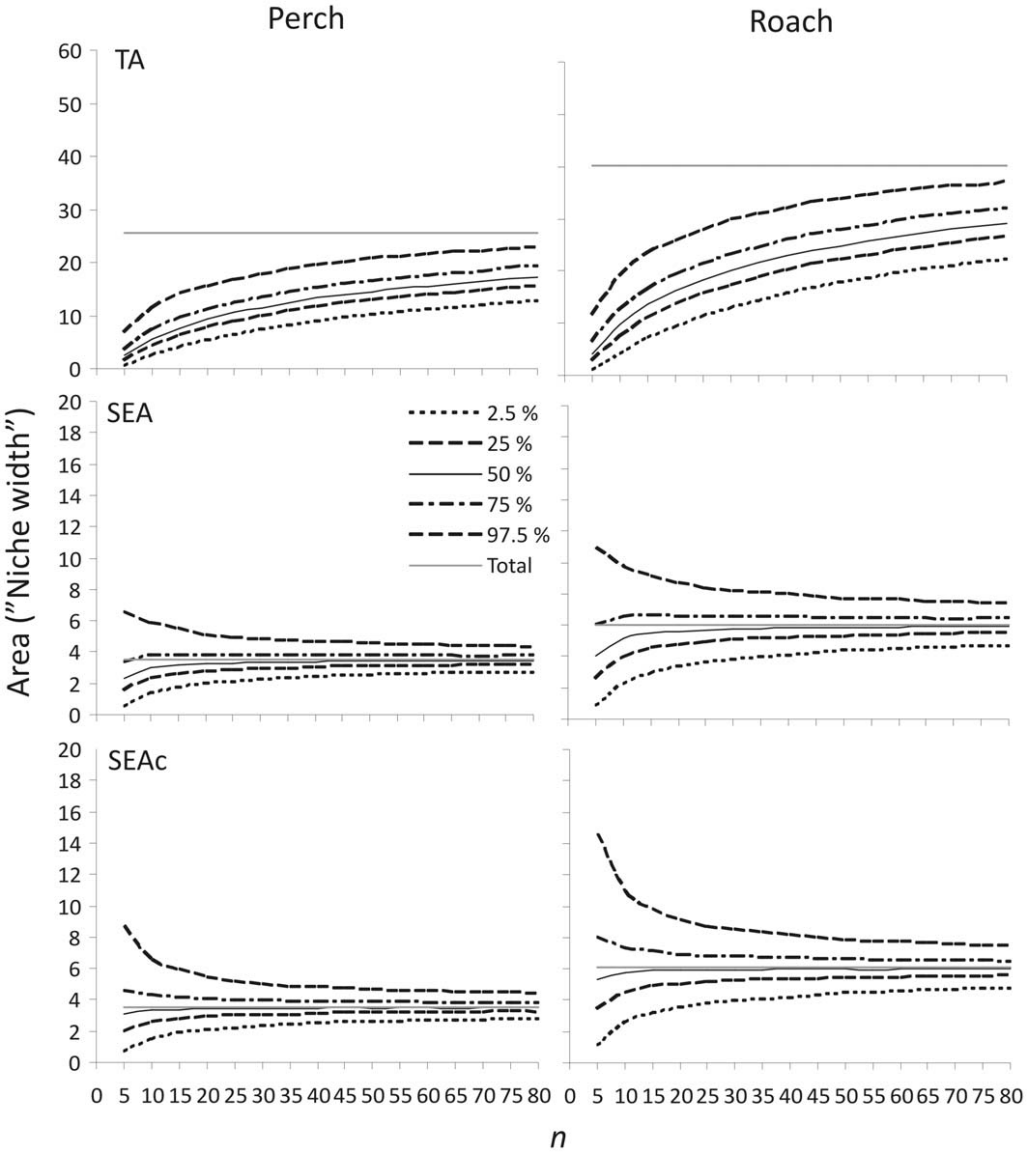
Isotopic niche widths calculated using simulated data. The data followed a multivariate normal distribution, but otherwise matched the empirical roach and perch isotope data with identical sample size, sample means and variance-covariance matrix. Lines represent the upper 97.5%, 75%, 50% and lower 25% and 2.5% percentiles for the niche area estimates after each 5000 resamplings with increasing sample size (*n*+5) from the simulated perch (left panel) and roach (right panel) populations.

Since the original data could not be transformed to follow a normal distribution, we simulated a new data set from the empirical perch and roach δ^13^C and δ^15^N data that matched the sample sizes, means and covariance matrices of the original datasets but followed a normal distribution. Repeating the analyses with these simulated data resulted in slightly better fits in the SEA and SEA_c_ metrics, and the median niche area values from 5000 resamplings were closer to the total observed niche area with lower (<30) sample sizes. This data transformation did not change the perch total SEA and SEA_c_ metric values (3.5), but decreased these from 6.7 to 6.1 for roach as some of the very high or low values were simulated as less extreme in the normalised data. However, even with the data following multivariate a normal distribution, the scatter of niche width observations with lower sample sizes (5–20) is considerable (Fig. 5). The SEA metric for perch with *n* = 15 ranged from 1.7 to 5.5 and SEA_c_ from 1.8 to 5.9 when 95% of observations are considered. Similarly the SEA and SEAc metrics for roach ranged from 3.0 to 9.1 and 3.2 to 9.8.

## Discussion

Our simple evaluation with an unusually large natural data set clearly illustrated how strongly sample size affected the estimates of observed population isotopic niche width, when estimated as a convex hull area (TA) in a δ^13^C–δ^15^N biplot. Moreover, the variation in our perch and roach population δ^13^C and δ^15^N values translated into wide percentile ranges, meaning that the sample sizes of typical SIA studies (very often less than 20 individuals, Fig. 1) would not provide reliable estimates of niche width. In fact, if we would compare population niche widths in Lake Jyväsjärvi with TA and sampled only 20 random individuals of perch and roach, by chance either of these species could be assigned a greater niche width (Fig. 4), when a much greater sample size would indicate roach occupying a wider niche [20] as is expected based on its more generalist feeding ecology.

This demonstration of the low accuracy of TA is important considering studies where different populations have been compared for their niche widths using TA in isotope biplots, without proper *a priori* knowledge of the variation associated with population δ^13^C and δ^15^N values. For example, Zambrano *et al.* [16] used TA from δ^13^C–δ^15^N biplots to estimate niche widths of a native axolotl (*Ambystoma maxicanum* Shaw 1789) and exotic carp (*Cyprinus carpio* L.) and tilapia (*Oreochromis niloticus* L.) in Mexico. They concluded that the native axolotl occupied the smallest niche area, followed by carp and tilapia. However, the sample sizes showed a similar pattern as axolotl clearly had the lowest and tilapia the highest number of individuals in these analyses. Similarly Darimont *et al.* [13] compared population isotopic niche areas of wolves (*Canis lupus* L.) from mainland and island habitats with different sample sizes (8, 24 and 46 individuals), and the smallest niche area was found for the population with the lowest number of observations and the largest area for the population with most observations. They attempted to compensate for the difference in sample size by resampling the larger populations using the same number of observations as in the smaller populations (10 000 resamplings from the larger populations) and comparing the iterated niche area distributions to the observed area of the smaller population. We argue that even though this approach may improve the comparability of results, the method is not fully valid, as the very low sample size in the smallest population is particularly hazardous. Judging from our data, the risk of not detecting the true population level isotopic variation, if only 8 individuals are sampled, is alarmingly high and can lead to significantly underestimated niche areas.

The standard ellipse areas proposed by Jackson *et al.* [18] clearly performed much better than the TA and were less dependent on sample sizes, particularly when considering at least 30 individuals from our data set. Nevertheless, and in contrast to the results of Jackson *et al.* [18] based on a simulated dataset with multivariate normal distribution, even the SEA_c_ could not completely remove the bias of underestimating the width in the case of low *n* in our empirical dataset where the distribution is skewed and variance is large. This underestimation was reduced when analysing our simulated data that fully followed a normal distribution. Neither SEA nor SEA_c_ metric could provide a very reliable niche width estimate with low sample size (*n*<30) when using empirical data (Fig. 4). The performance was slightly improved with simulated data (Fig. 5) but lower samples sizes still contained major uncertainties. This highlights that the required sample size for accurate analyses is largely dependent on the observed isotope range in a consumer population, which itself is largely dictated by the range in prey isotope values. While the random error dropped to a steady level with *n*>30, 95% of the estimated niche widths still varied between the observed value ±40–50%, and 50% of estimated niche widths varied between the observed value ±10–25% in the empirical data. Jackson *et al.* [18] recommended a minimum of 10 samples per group as the smallest reliable sample size but this seems to be too low according to our data sets (empirical and simulated). For example, with just 10 roach individuals from Jyväsjärvi, it would be just as likely to first obtain a SEA value of 3.5 but then 7.1 with the next set of 10 roach individuals. Similarly the SEA_c_ value could equally well be 3.9 or 8.0. The percentile values are of course products of the variation and frequency distribution of the δ^13^C and δ^15^N values in the analysed dataset. Studying populations with less variation would presumably result in more precise niche width estimates, even with slightly lower sample sizes. Unfortunately it is rarely easy to know what the real isotope distributions or ranges are at the population level if only a small number of individuals have been analysed. Hence, low sample sizes should be interpreted with great caution when estimating isotopic niche width, at least without strong evidence of very low population level variation in δ^13^C and δ^15^N values. Nevertheless, given sufficient sample sizes and low isotope variation, the SEA method (SIBER) certainly offers a more robust method than TA to estimate and compare population isotope niche widths.

An alternative to using TA calculated from all analysed individuals from a population is to use only the most densely “clustered” area of individuals in a δ^13^C–δ^15^N biplot. This could be done for example by calculating the convex hull area for 95% of individuals, thereby excluding the more divergent observations that quickly expand the estimated TA [24]. However, the value of niche width estimates derived from only part of the population is highly questionable as the overall population niche is made up of individuals' niches within that population. Hence use of a metric value that does not include all individuals in a population risks ignoring important information on intraspecific variation that should be considered when describing the niche of a population, particularly considering the growing evidence for intraspecific niche variation and individual diet specialisation [25].

It is also crucial to remember that these isotope niche areas cannot be directly compared among different habitats or ecosystems without accounting for isotope variation in the resource or prey items (i.e. baselines). This is analogous to estimating consumer trophic positions using δ^15^N values where values from two different habitats cannot be directly compared to each other without accounting for baseline level δ^15^N differences. Similarly the range in population δ^13^C and δ^15^N values, defining the TA and SEA, is ultimately driven by the variation and range in δ^13^C and δ^15^N values of their prey and hence the ecosystem will largely control the isotope space [11],[12]. While such variation and range might be smaller in some cases, such as many terrestrial systems, lake ecosystems for example can often show considerable differences between pelagic and littoral prey isotope values within the same lake. Therefore a fish population utilising both littoral and pelagic prey sources in one lake can generate much smaller isotopic niche areas than a population of conspecifics in another lake, even though feeding on exactly the same prey and utilising similar habitats, simply because the variation in prey δ^13^C and δ^15^N differs between the lakes. This also means that the isotope niche should be properly separated from ecological trophic niche, since only one fraction of isotope niche reflects the true ecological trophic niche.

When populations in different habitats or ecosystems are to be compared for their isotope niche widths, corrections for standardising the basal variation in δ^13^C and δ^15^N values need to be made. One method was proposed by Olsson *et al.* [14] who standardised the carbon isotope values of consumers using the mean and range of δ^13^C of their prey. Another potential method is to use mixing models [26],[27] to source contributions, such as littoral or pelagic reliance, and use those values instead of the original δ^13^C [28],[15],[29]. The method proposed by Newsome *et al.* [28] transforms the typical δ-space into p-values calculated from source proportions, which can then be used to calculate niche widths with more common metrics, such as the Shannon-Wiener measure [28]. The δ^15^N values can also be standardised by calculating trophic positions for consumers following [30], where the potential variation in basal δ^15^N values is incorporated into the estimate of trophic position, and these values can then be used instead of the original δ^15^N values [14],[15]. Combining trophic positions and source p-values in the TA or SEA method could provide an interesting alternative [15] but would be limited to studies with clearly defined sources.

In conclusion, our results clearly show how increasing sample size dramatically affects the observed isotopic niche area estimated as a convex hull TA. Also, a random draw of e.g. 10–15 individuals from our natural perch and roach populations resulted in 5–10 fold differences in the observed isotopic niche areas (for 95% of observations). Therefore we agree with Jackson *et al.* [18] that TA should not be used to estimate population niche widths from stable isotope data. The SEA method was generally less sensitive to sample size and evidently is a much more robust method to assess isotopic niche widths. We certainly recommend use of SEA instead of TA for evaluation of population niche widths. However, the SEA method still suffered from appreciable uncertainty, particularly with sample sizes <30 individuals, in our empirical dataset with skewed distributions. Uncertainty was reduced when the method was applied to a simulated multivariate normal data set, but since many actual populations are likely to be skewed like our empirical data, and the “outlier” individuals may be particularly interesting and important for the adaptation of populations, the result highlights the need to consider carefully the applicability of such methods as a measure of trophic niche widths of consumers without adequate sample sizes and if population level isotope variation is not well established *a priori*. Reliability of the SEA could be increased by using greater sample sizes (>30 individuals) and by applying a bootstrapped resampling of for example 10 individuals from each population. This allows the comparison of median niche width values among populations, further increasing the robustness of the SEA method. Based on the data for published studies presented in Fig. 1, we argue that too many current studies are relying on sample sizes that are too small to provide robust conclusions about niche widths, and we urge persons contemplating such studies to incorporate sample sizes of at least n = 30 into their research plans wherever possible.

## Supporting Information

Appendix S1 **Reference list for publications used for** **Fig. 1** **.** The data were sourced through literature search in ISI Web of Knowledge and Scopus.
(DOC)
