# Supplementary material for: An Empirical Evaluation of the Utility of Convex Hull and Standard Ellipse Areas for Assessing Population Niche Widths from Stable Isotope Data
Source: PLoS One. 2013 Feb 6;8(2):e56094. doi: 10.1371/journal.pone.0056094 (PMC3566058; doi:10.1371/journal.pone.0056094)
Supplement: Appendix S1 — Reference list for publications used for Fig. 1 . The data were sourced through literature search in ISI Web of Knowledge and Scopus. (DOC) [file pone.0056094.s001.doc]

Blanco-Fontao B, Obeso JR, Bañuelos M-J, Quevedo M (2012) Habitat partitioning and molting site fidelity in *Tetrao urogallus cantabricus* revealed through stable isotopes analysis. J Ornithol 153: 555−562.

Dalerum F, Perbro A, Magnusdottir R, Hersteinsson P, Angerbjörn A (2012) The influence of coastal access on isotope variation in Icelandic Arctic foxes. PLoS ONE 7: e32071.

Darimont CT, Paquet PC, Reimchen, TE (2009) Landscape heterogeneity and marine subsidy generate extensive intrapopulation niche diversity in a large terrestrial vertebrate. J Anim Ecol 78: 126–133.

Durbec M, The BN, Grey J, Harrod C, Stolzenberg N et al. (2010) Biological influences on inter- and intraspecific isotopic variability among paired chondrostome fishes. CR Biol 333: 613–621.

Eloranta AP, Siwertsson A, Knudsen R, Amundsen P-A (2011) Dietary plasticity of Arctic charr (*Salvelinus alpinus*) facilitates coexistence with competitively superior European whitefish (*Coregonus lavaretus*). Ecol Freshw Fish 20: 558–568.

Franco-Trecu V, Aurioles-Gamboa D, Arim M, Lima, M (2012) Prepartum and postpartum trophic segregation between sympatrically breeding female Arctocephalus australis and *Otaria flavescens.* J Mammal 93: 514−521.

Grey J, Jackson MC (2012) ‘Leaves and Eats Shoots’: Direct terrestrial feeding can supplement invasive red swamp crayfish in times of need. PLoS ONE 7: e42575.

Jackson MC, Donohue I, Jackson AL, Britton JR, Harper DM et al. (2012) Population-level metrics of trophic structure based on stable isotopes and their application to invasion ecology. PLoS ONE 7: e31757.

Kadye WT, Booth AJ An invader within an altered landscape: One catfish, two rivers and an inter-basin water transfer scheme. River Res Applic In press.

Klarner B, Maraun M, Scheu S Trophic diversity and niche partitioning in a species rich predator guild − Natural variations in stable isotope ratios (^13^C/^12^C, ^15^N/^14^N) of mesostigmatid mites (Acari, Mesostigmata) from Central European beech forests. Soil Biol Biochem In press.

Layman CA, Quattrochi JP, Peyer CM, Allgeier JE (2007) Niche width collapse in a resilient top predator following ecosystem fragmentation. Ecol Lett 10: 937−944.

Layman CA, Allgeier JA (2012) Characterizing trophic ecology of generalist consumers: a case study on the invasive lionfish Pterois volitans in The Bahamas. Mar Ecol Prog Ser 448: 131−141.

McHugh P, McIntosh A, Howard S, Budy P Niche flexibility and trout-galaxiid co-occurrence in a hydrologically diverse riverine landscape. Biol Invasions In press.

Mercado-Silva N, Helmus MR, Vander Zanden JM (2009) The effects of impoundment and non-native species on a river food web in Mexico’s central plateau. River Res Applic 25: 1090–1108.

Olsson K, Stenroth P, Nyström P, Granéli W (2009) Invasions and niche width: does niche width of an introduced crayfish differ from a native crayfish? Freshwat Biol 54: 1731–1740.

Quevedo M, Svanbäck R, Eklöv P (2009) Intrapopulation niche partitioning in a generalist predator limits food web connectivity. Ecology 90: 2263–2274.

Robb GN, Woodborne S, Bennett NC (2012) Subterranean Sympatry: An Investigation into Diet Using Stable Isotope Analysis. PLoS ONE 7: e48572

Ruokonen TJ, Karjalainen J, Kiljunen M, Pursiainen M, Hämäläinen H (2012) Do introduced crayfish affect benthic fish in stony littoral habitats of large boreal lakes? Biol Invasions 14: 813−825.

Sellanes J, Zapata-Hernández G, Pantoja S, Jessen GL (2011) Chemosynthetic trophic support for the benthic community at an intertidal cold seep site at Mocha Island off central Chile Estuar Coast Shelf Sci 95: 431−439.

Semmens BX, Ward EJ, Moore JW, Darimont CT (2009) Quantifying inter- and intra- population niche variability using hierarchical Bayesian stable isotope mixing models. PLoS ONE 4: e6187.

Swanson HK, Kidd KA, Reist JD (2010) Effects of Partially Anadromous Arctic Charr (*Salvelinus alpinus*) Populations on Ecology of Coastal Arctic Lakes. Ecosystems 11: 261−274.

Tarroux A, Bêty J, Gauthier G, Berteaux D (2012) The marine side of a terrestrial carnivore: Intra-population variation in use of allochthonous resources by Arctic foxes. PLoS ONE 7: e42427.

Thomson JA, Heithaus MR, Burkholder DA, Vaudo JJ, Wirsing AJ et al. (2012) Site specialists, diet generalists? Isotopic variation, site fidelity, and foraging by loggerhead turtles in Shark Bay, Western Australia. Mar Ecol Prog Ser 453: 213−226.

Toscano, BJ, Pulcini D, Hayden B, Russo T, Kelly-Quinn M et al. (2010) An ecomorphological framework for the coexistence of two cyprinid fish and their hybrids in a novel environment. Biol J Linn Soc 99: 768−783.

Zambrano L, Valiente E, Vander Zanden MJ (2010) Food web overlap among native axolotl (*Ambystoma mexicanum*) and two exotic fishes: carp (*Cyprinus carpio*) and tilapia (*Oreochromis niloticus*) in Xochimilco, Mexico City. Biol Invasions 12: 3061–3069.
